# Quantum iterative reconstruction on a photon-counting detector CT improves the quality of hepatocellular carcinoma imaging

**DOI:** 10.1186/s40644-023-00592-5

**Published:** 2023-07-21

**Authors:** Dirk Graafen, Fabian Stoehr, Moritz C. Halfmann, Tilman Emrich, Friedrich Foerster, Yang Yang, Christoph Düber, Lukas Müller, Roman Kloeckner

**Affiliations:** 1 Diagnostic and Interventional Radiology, University Medical Center of the Johannes Gutenberg-University Mainz, Mainz, Germany; 2 German Center for Cardiovascular Research (DZHK), Partner-Site Rhine-Main, Mainz, Germany; 3 Division of Cardiovascular Imaging, Department of Radiology and Radiological Science, Medical University of South Carolina, Charleston, SC USA; 4 Department of Medicine I, University Medical Center of the Johannes Gutenberg-University Mainz, Mainz, Germany; 5 Present Address: Institute of Interventional Radiology, University Hospital Schleswig-Holstein, Campus Lübeck, Lübeck, Germany

**Keywords:** Photon-counting detector CT, Hepatocellular carcinoma, Quantum iterative reconstruction

## Abstract

**Background:**

Excellent image quality is crucial for workup of hepatocellular carcinoma (HCC) in patients with liver cirrhosis because a signature tumor signal allows for non-invasive diagnosis without histologic proof. Photon-counting detector computed tomography (PCD-CT) can enhance abdominal image quality, especially in combination with a novel iterative reconstruction algorithm, quantum iterative reconstruction (QIR).

The purpose of this study was to analyze the impact of different QIR levels on PCD-CT imaging of HCC in both phantom and patient scans.

**Methods:**

Virtual monoenergetic images at 50 keV were reconstructed using filtered back projection and all available QIR levels (QIR 1–4). Objective image quality properties were investigated in phantom experiments. The study also included 44 patients with triple-phase liver PCD-CT scans of viable HCC lesions. Quantitative image analysis involved assessing the noise, contrast, and contrast-to-noise ratio of the lesions. Qualitative image analysis was performed by three raters evaluating noise, artifacts, lesion conspicuity, and overall image quality using a 5-point Likert scale.

**Results:**

Noise power spectra in the phantom experiments showed increasing noise suppression with higher QIR levels without affecting the modulation transfer function. This pattern was confirmed in the in vivo scans, in which the lowest noise levels were found in QIR-4 reconstructions, with around a 50% reduction in median noise level compared with the filtered back projection images. As contrast does not change with QIR, QIR-4 also yielded the highest contrast-to-noise ratios. With increasing QIR levels, rater scores were significantly better for all qualitative image criteria (all *p* < .05).

**Conclusions:**

Without compromising image sharpness, the best image quality of iodine contrast optimized low-keV virtual monoenergetic images can be achieved using the highest QIR level to suppress noise. Using these settings as standard reconstruction for HCC in PCD-CT imaging might improve diagnostic accuracy and confidence.

## Background

Hepatocellular carcinoma (HCC) is one of the most common cancers worldwide with increasing incidence and a high rate of cancer-related deaths [1, 2]. Cross-sectional imaging plays a pivotal role in the diagnosis of HCC [3], which can be achieved with high certainty based on characteristic tumor signal behavior in contrast-enhanced cross-sectional imaging. Thus, in patients with liver cirrhosis and this characteristic signal behavior, including arterial phase hyperenhancement and non-peripheral washout, diagnosis of HCC based solely in imaging is possible, without need for further histologic proof [3, 4]. Achieving good imaging, however, requires high-quality standards for the imaging methods used. Establishing these standards can enable accurate diagnosis while also improving treatment allocation, prognosis prediction, and tumor response assessment, especially with the use of promising imaging biomarkers such as delayed percentage attenuation ratio [3, 5].

Recently, a novel computed tomography (CT) scanner that uses photon-counting detectors (PCDs) was approved for clinical use. These semiconductor crystal detectors directly convert X-ray photons into electric signals, enabling the measurement of single photons and their corresponding energies [6–8]. PCD-CT improves dose efficiency and spatial resolution, reduces image noise, increases contrast-to-noise ratios (CNRs), and inherently provides spectral data especially including but not limited to abdominal scans [9–19].

Image reconstruction parameters, including characteristics of the applied convolution kernel and iterative reconstruction, influence CT image quality [20]. As a spectral CT method comparable to dual-energy CT, PCD-CT allows for calculation of virtual monoenergetic images (VMIs), which involves the addition of their keV levels as a factor affecting image quality. A few studies have demonstrated the benefit of low-keV VMIs for late arterial and for portal venous phase abdominal PCD-CT [18, 21, 22]. The PCD-CT scanner was introduced with a novel iterative reconstruction algorithm known as quantum iterative reconstruction (QIR). Recently, the benefits of this algorithm were shown for abdominal PCD-CT [23], but the focus of that work was limited to the portal venous phase in an inhomogeneous patient cohort with only one case of HCC.

To place the focus on HCC, we investigated the diagnostic potential of PCD-CT with QIR for triple-phase HCC imaging, using in vitro phantom experiments and in vivo image analysis.

## Methods

### Imaging protocols

PCD-CT scans were performed with a first-generation Naeotom Alpha® scanner (Siemens Healthineers, Erlangen, Germany). All images were reconstructed as VMIs at 50 keV, which was in the optimal keV level range identified for abdominal PCD-CT scans in the late arterial and portal venous phase in previous studies [18, 21, 22]. As recommended in Ref. [24], the soft quantitative kernel Qr36 was applied. Images were reconstructed using filtered back projection (FBP = QIR-0) and all available iterative reconstruction levels (QIR-1 to QIR-4). In a service pack 1 update of software version VA40A, a new higher QIR level was introduced, and the nomenclature was shifted by 1, so that the old QIR-2 level was renamed QIR-1, the old QIR-3 was renamed QIR-2, and so on. The old QIR-1 level is no longer available and is given as QIR-# in the current work. Detailed information about acquisition and reconstruction parameters is summarized in Table 1.

**Table 1:** Technical data for the CT protocol and image reconstruction parameters

Software version	Syngo CT VA40A until March 2022 Syngo CT VA40A sp1 since April 2022
Single collimation	0.4 mm
Total collimation	57.6 mm
Tube voltage	120 kVp
Iterative reconstruction	FBP and all QIR levels
Convolution kernel	Qr36
Slice thickness	1 mm
Tube current modulation	CARE Dose4D

*FBP* Filtered back projection, *QIR* Quantum iterative reconstruction

### Phantom measurement

Phantom experiments were performed to determine the modulation transfer functions (MTFs) and noise power spectra (NPS) of the different QIR levels.

#### Modulation transfer function

MTFs were determined with the wire phantom method as previously described [25], using a quality test phantom with a thin wire 5 cm in length. The phantom was measured using a fixed tube current with an effective tube current exposure time product of 380 mAs (CTDI_vol_ = 30 mGy). Minimum possible voxel sizes of the QuantumPlus acquisition mode were reconstructed, i.e., a slice thickness of 0.4 mm and a pixel spacing of 0.049 mm.

#### Noise power spectrum

A cylindrical segment of a water phantom with a length of 10 cm and a diameter of 30 cm was measured for determining the NPS of the available QIR levels. The phantom was measured using a fixed tube current with an effective tube current exposure time product of 190 mAs (CTDI_vol_ = 15 mGy). Images were reconstructed that were identical to patient images with a slice thickness of 1 mm and a pixel spacing of 0.77 mm. Subtraction of two consecutive acquisitions with identical acquisition parameters provided noise images, as described previously [26]. Both MTFs and NPS were calculated using the computing platform MATLAB (version R2021b, The MathWorks, Inc., Natick, MA, USA).

### Study population

From February to May 2022, a total of 78 consecutive patients with either suspected or confirmed HCC were scanned on a PCD-CT for imaging evaluation of the liver. Prospectively, these scans were reconstructed with the above mentioned variation of QIR level. Of this group, 34 patients were excluded for a lack of viable HCC lesions with the characteristic signal behavior. Thus, a total of 44 patients with viable HCC lesions showing late arterial phase hyperenhancement and washout in the delayed phase were identified and included in this study.

### Contrast media protocol

The injection protocol consisted of a single-bolus contrast media injection (120 ml volume, 5 ml/s flow, 1.9 gI/s iodine flux; Ultravist® 370, Bayer Vital, Leverkusen, Germany) followed by a saline bolus (50 ml volume, 4 ml/s flow). Timing of the late arterial phase was achieved by bolus tracking in the proximal abdominal aorta with a threshold of 100-HU signal increase and 13-s post-threshold delay. The portal venous phase and delayed phase were acquired with a delay of 50 s and 180 s, respectively.

### Quantitative image analysis

A board-certified consultant radiologist with 15 years of experience and a resident with 4 years of experience performed quantitative analysis of the images. Mean attenuation was measured by regions of interest (ROIs). Liver attenuation was calculated by averaging the mean attenuation of three circular liver ROIs with a diameter of 1 – 2 cm placed in the left lateral section, in the left medial or right anterior section, and in the right posterior section. Mean vascular attenuations were determined as follows. For the late arterial phase, the mean attenuation was determined for the aorta at the level of the celiac trunk, as well as for the proximal right and left hepatic arteries. For the portal venous phase, the mean attenuation was determined for the aorta, the proximal right and left hepatic arteries, and the main, right, and left portal veins. For the delayed phase, the mean attenuation was determined for the main and left and right portal veins, along with the dominant hepatic artery and inferior vena cava.

Image noise was determined based on the standard deviation of two ROIs placed in the left and right erector spinae muscles at the level of the ostium of the celiac trunk. Contrasts were calculated for the intrahepatic HCC lesions and for the vascular attenuation in relation to the liver attenuation using the following formulas:

$${\text{C}}_{\text{lesion}}={\text{lesion attenuation}}-\text{liver attenuation}$$

$${\text{C}}_{\text{vascular}}=\text{mean vascular attenuation}-\text{liver attenuation}$$

All measurements were performed with the image processing and analysis software ImageJ (version 1.53) [27]. ROIs were defined in one of the eight reconstructed images and stored in the program’s ROI manager. A macro was created to automatically perform the mean attenuation measurements in these ROIs for all eight images, i.e., the eight different convolution kernels, to ensure measurements were being taken at exactly the same position.

### Qualitative image analysis

Three readers evaluated the image quality — one board-certified consultant radiologist with 15 years of experience and two residents with respectively 3 and 4 years of experience in abdominal CT imaging. For every patient and each contrast phase, all five reconstructed images (FBP and QIR-1 to QIR-4) were presented in a randomly ordered 2- × -3 side-by-side arrangement using the institutional picture archiving and communication system (PACS: Sectra®, Linköping, Sweden) blinded to image information. Standard abdominal windowing settings were used (W:400, L:60) with the option to change the windowing settings separately for every image.

Analogous to previous studies [18, 21, 23, 28, 29], the following quality criteria were assessed using a 5-point Likert scale: image noise (0 = very strong, 1 = strong, 2 = moderate, 3 = little, 4 = no/very little); image artifacts and diagnostic confidence (0 = severe artifacts, non-diagnostic; 1 = severe artifacts, confidence degraded, diagnosis questionable; 2 = moderate artifacts, decreased confidence but diagnosis still possible; 3 = mild artifacts, no change in confidence; 4 = no artifacts, high diagnostic confidence); and overall image quality (0 = non-diagnostic, 1 = bad, 2 = moderate, 3 = good, 4 = excellent). For evaluation of lesion conspicuity, a comparative scale was used, with the worst image rated as 0 and the other images rated on the following scale: 0 = similar to worst reconstruction, 1 = slightly better/no influence on diagnosis, 2 = mildly better/possible influence on diagnosis, 3 = moderately better/probable influence on diagnosis, and 4 = markedly better/improved diagnosis.

### Statistical analysis

All statistical analyses were executed with dedicated statistical software (R, version 4.1.1, R Foundation for Statistical Computing, Vienna, Austria). Categorical and binary baseline parameters are reported as absolute numbers and percentages and ordinal-scaled variables as medians and interquartile ranges. Interval-scaled, normally distributed variables, based on the Shapiro–Wilk test, are reported as means and standard deviations. Statistical differences in quantitative and qualitative image parameters were analyzed using the paired samples Wilcoxon rank test with Bonferroni correction for multiple comparisons. *P* values < 0.05 were considered statistically significant.

Krippendorff’s alpha was used for inter-reader agreement testing with the following interpretation of the alpha value: 0.0–0.2 slight agreement, 0.2–0.4 fair agreement, 0.4–0.6 moderate agreement, 0.6–0.8 substantial agreement, and 0.8–1.0 near-perfect agreement [18, 21].

## Results

### Phantom measurements

**Fig. 1:**
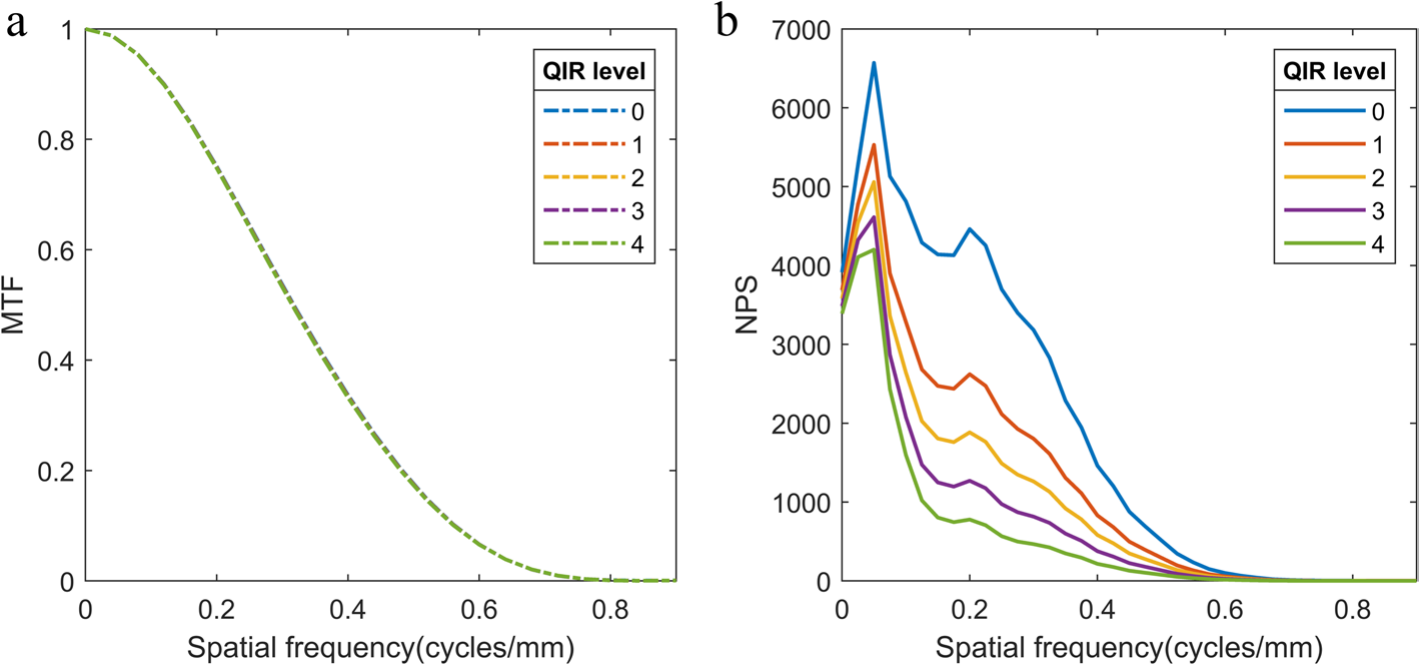
Modulation transfer functions (MTF) and noise power spectra (NPS) of the quantum iterative reconstruction (QIR) levels. Level 0 shows the results for the filtered back projection reconstruction. Caused by the almost perfect overlap, the MTFs of QIR level 0 – 2 are not visible

MTFs and NPS of the different QIR levels are shown in Fig. 1. The QIR algorithm shows no relevant effect on the MTF. Increasing QIR levels led to more pronounced suppression of noise power. Noise magnitude ratios [30] were 67% for QIR-1, 54% for QIR-2, 42% for QIR-3, and 33% for QIR-4. Central frequencies of the NPS shifted to lower values (FBP: 0.19 mm^−1^; QIR-1: 0.17 mm^−1^; QIR-2: 0.15 mm^−1^; QIR-3: 0.13 mm^−1^; and QIR-4: 0.11 mm^−1^), resulting in central frequency ratios of 89% for QIR-1, 81% for QIR-2, 71% for QIR-3, and 59% for QIR-4.

### Baseline characteristics and radiation dose

In the images of the 44 included patients, a total of 75 viable HCC lesions were evaluated in the quantitative image analysis (22 patients with one, 13 patients with two, and 9 patients with three evaluated lesions). Table 2 lists the baseline characteristics and radiation doses. In the portal venous phase, 26 patients received an abdominal scan only, 3 patients a scan of the abdomen and pelvis, and 15 patients a scan of the thorax, abdomen, and pelvis.

**Table 2:** Baseline characteristics and radiation dose

Patient number	*N* = 44
Age (y)	67 (64–74)
Sex	
Female	13 (30%)
Male	31 (70%)
Body height (cm)	175 (169–178)
Body weight (kg)	79 (65–86)
Body mass index (kg/m^2^)	25.6 (23.1–28.0)
Late arterial phase	
CTDI_vol_ (mGy)	14 (12–16)
DLP (mGy*cm)	400 (300–450)
Effective dose (mSv)	6.1 (4.6 – 6.8)
Portal venous phase	Abd / Abd + Pel / Th + Abd + Pel
CTDI_vol_ (mGy)	14 (12–17) / 12 (12–12) / 16 (13–18)
DLP (mGy*cm)	370 (300–450) / 590 (540–630) / 1100 (890–1190)
Effective dose (mSv)	6.1 (4.4 – 6.8) / 8.7 (8.1 – 9.4) / 15.7 (13.3 – 17.8)
Delayed phase	
CTDI_vol_ (mGy)	14 (11–16)
DLP (mGy*cm)	400 (300–450)
Effective dose (mSv)	6.1 (4.5 – 6.8)

Unless otherwise indicated, medians are shown with interquartile ranges in parentheses

### Quantitative image analysis

**Fig. 2:**
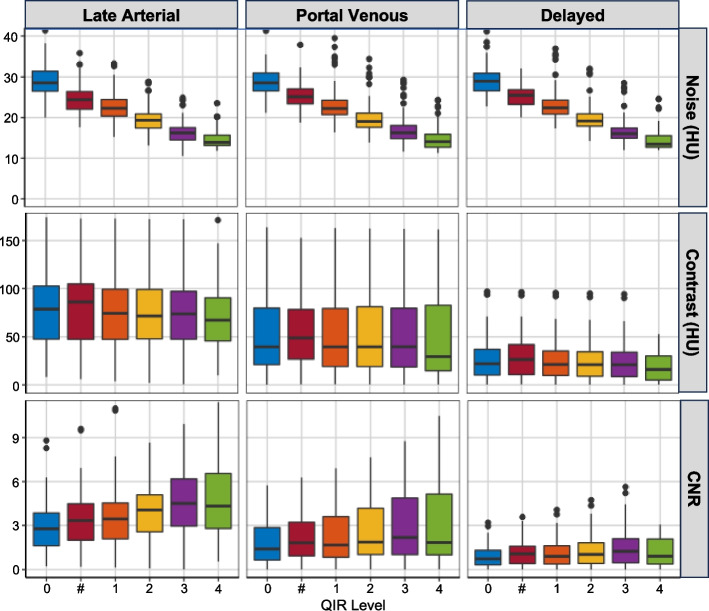
Dependence of image noise, HCC lesion-to-liver contrast, and contrast-to-noise ratio (CNR) on the respective quantum iterative reconstruction (QIR) levels. Level 0 shows the values of the filtered back projection

In all contrast phases, noise levels decreased significantly with higher QIR levels (all *p* < 0.05, Fig. 2, Table 3). The highest QIR level (QIR-4) yielded a noise level reduction of around 50% in comparison with FBP (QIR-0).

**Table 3:** Quantitative analyses of image noise and vascular and HCC Lesion Contrast-to-Noise Ratios (CNRs)

QIR level	0	#	1	2	3	4
**Late arterial**						
**Noise (HU)**	29 (27–31)		22 (20–24)	19 (17–21)	16 (14–18)	
	27 (25–30)	24 (22–26)	21 (19–23)	18 (17–20)	15 (14–17)	
	31 (29–32)		24 (23–25)	20 (20–22)	17 (16–19)	14 (13–16)
**CNR**_**vascular**_	11 (6–18)		14 (8–23)	17 (10–26)	19 (11–32)	
	12 (6–20)	14 (7–23)	17 (9–26)	19 (12–29)	24 (13–38)	
	9 (6–16)		13 (8–20)	15 (9–23)	15 (10–26)	22 (12–33)
**CNR**_**HCC**_	2.8 (1.6–3.8)		3.4 (2.1–4.5)	4.1 (2.6–5.1)	4.5 (3.0–6.2)	
	3.0 (1.8–4.0)	3.3 (2.0–4.5)	3.6 (2.3–4.9)	4.4 (2.7–5.5)	5.1 (3.2–6.8)	
	2.1 (1.3–3.2)		2.7 (1.8–4.0)	3.0 (2.0–4.6)	3.6 (2.8–5.5)	4.3 (2.8–6.5)
**Portal venous**						
**Noise (HU)**	29 (27–31)		22 (21–24)	19 (18–21)	16 (15–18)	
	28 (26–30)	25 (23–27)	22 (20–24)	19 (17–20)	16 (14–18)	
	29 (27–32)		23 (21–25)	20 (18–22)	16 (15–19)	14 (13–16)
**CNR**_**vascular**_	7 (5–8)		8 (6–10)	10 (7–12)	11 (8–14)	
	7 (5–9)	8 (6–10)	9 (7–11)	11 (8–13)	13 (10–16)	
	6 (4–7)		7 (6–9)	8 (7–11)	9 (8–12)	11 (9–15)
**CNR**_**HCC**_	1.4 (0.7–2.9)		1.6 (0.8–3.6)	1.9 (1.0–4.2)	2.2 (1.0–4.9)	
	1.6 (0.9–2.9)	1.8 (0.9–3.2)	2.1 (1.1–3.6)	2.5 (1.3–4.2)	2.9 (1.5–5.0)	
	0.9 (0.5–2.6)		1.1 (0.6–3.3)	1.3 (0–7–3.8)	1.5 (0.8–4.4)	1.8 (1.0–5.1)
**Delayed**						
**Noise (HU)**	28 (27–31)		22 (21–24)	19 (18–21)	16 (15–17)	
	29 (26–30)	25 (23–27)	22 (20–24)	19 (18–20)	16 (14–17)	
	29 (27–33)		23 (21–26)	20 (18–22)	16 (16–19)	13 (13–16)
**CNR**_**vascular**_	2.2 (1.7–3.0)		2.7 (2.1–3.8)	3.2 (2.3–4.4)	3.6 (2.8–5.2)	
	2.3 (1.8–3.0)	2.6 (2.0–3.3)	2.9 (2.3–3.8)	3.4 (2.5–4.4)	4.1 (3.0–5.2)	
	2.1 (1.5–3.0)		2.7 (1.8–3.9)	3.1 (2.1–4.2)	3.6 (2.5–5.0)	4.2 (3.0–6.0)
**CNR**_**HCC**_	0.7 (0.3–1.3)		0.9 (0.4–1.6)	1.0 (0.4–1.8)	1.2 (0.5–2.1)	
	1.0 (0.4–1.4)	1.1 (0.4–1.6)	1.2 (0.5–1.8)	1.3 (0.6–2.1)	1.6 (0.6–2.4)	
	0.5 (0.2–1.2)		0.6 (0.2–1.4)	0.7 (0.3–1.6)	0.8 (0.3–1.8)	0.9 (0.4–2.1)

Values are medians with interquartile ranges in parentheses. For each, in the top line, the results for the total study population are reported. The middle and bottom lines depict the results for the populations scanned before and after the service pack installation

The iodine contrast was not affected by the QIR algorithm (Fig. 2), and the reduction in noise yielded increased CNRs for the HCC lesions and the vessels. Thus, CNRs in the images reconstructed with the highest QIR level (QIR-4) were approximately twice the CNRs in the FBP reconstructions. However, for the CNR of the lesions, only the difference in the two highest QIR levels (QIR-3 and QIR-4) from the FBP reached statistical significance (*p* < 0.05).

### Qualitative image analysis

**Fig. 3:**
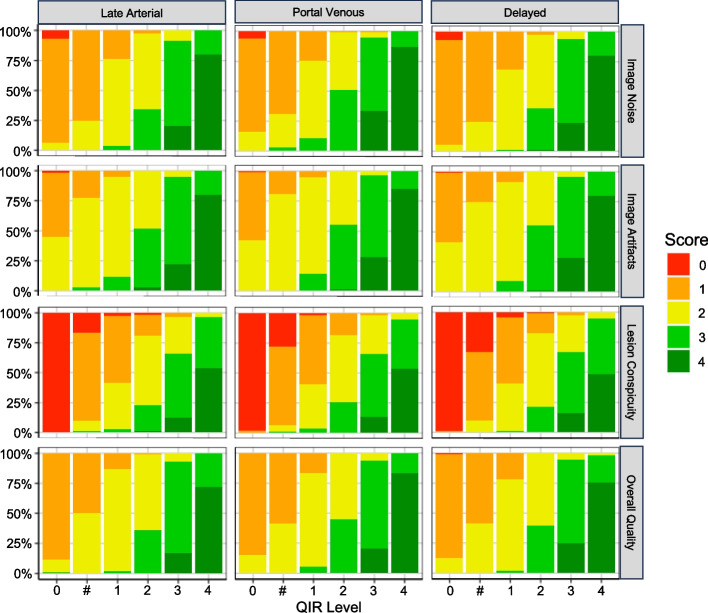
Qualitative image analyses of image noise, image artifacts, lesion conspicuity, and overall image quality of the different contrast phases of the triple-phase HCC examination protocol

Results of the qualitative image analysis are illustrated in Fig. 3 and Table 4. In all three contrast phases of the triple-phase liver protocol, inter-rater reliability was near perfect (late arterial phase: alpha = 0.81; portal venous phase: alpha = 0.83; and delayed phase: alpha = 0.82). All three raters found in all three contrast phases that the strong image noise and severe artifacts with degraded confidence (score 1) in the FBP images were increasingly reduced with increasing QIR level, resulting in very little noise without artifacts and with high diagnostic confidence (score 4) in the QIR-4 images.

**Table 4:** Median scores of qualitative image analyses

Quality criteria	FBP	QIR-#	QIR-1	QIR-2	QIR-3	QIR-4
**Late arterial phase**						
Image noise	1 (1–1)	1 (1–1)	2 (2–2)	2 (2 –3)	3 (3–3)	4 (4–4)
Image artifacts	1 (1–2)	2 (2–2)	2 (2–2)	3 (2–3)	3 (3–3)	4 (4–4)
Lesion conspicuity	0 (0 –0)	1 (1–1)	1 (1–2)	2 (2–2)	3 (2–3)	4 (3–4)
Overall image quality	1 (1–1)	1.5 (1 –2)	2 (2–2)	2 (2–3)	3 (3–3)	4 (3–4)
**Portal venous phase**						
Image noise	1 (1–1)	1 (1–2)	2 (2–2)	3 (2–3)	3 (3–4)	4 (4–4)
Image artifacts	1 (1–2)	2 (2–2)	2 (2–3)	3 (2–3)	3 (3–4)	4 (4–4)
Lesion conspicuity	0 (0–0)	1 (0–1)	1 (1–2)	2 (2–3)	3 (2–3)	4 (3–4)
Overall image quality	1 (1–1)	1 (1–2)	2 (2–2)	2 (2–3)	3 (3–3)	4 (4–4)
**Delayed phase**						
Image noise	1 (1–1)	1 (1–1)	2 (1–2)	2 (2–3)	3 (3–3)	4 (4–4)
Image artifacts	1 (1–2)	2 (1.25–2)	2 (2–2)	3 (2–3)	3 (3–4)	4 (4–4)
Lesion conspicuity	0 (0–0)	1 (0–1)	1 (1–2)	2 (2–2)	3 (2–3)	3 (3–4)
Overall image quality	1 (1–1)	1 (1–2)	2 (2–2)	2 (2–3)	3 (3–3.25)	4 (4–4)

Interquartile ranges are presented in parentheses

The raters also assessed a relevant improvement in lesion conspicuity. The image changes for low QIR levels yielded little to no improvement in diagnosis (score 1–2), but the higher QIR levels yielded markedly better scores with a probable influence on diagnosis (score 3) or even subjectively improved diagnosis (score 4).

**Fig. 4:**
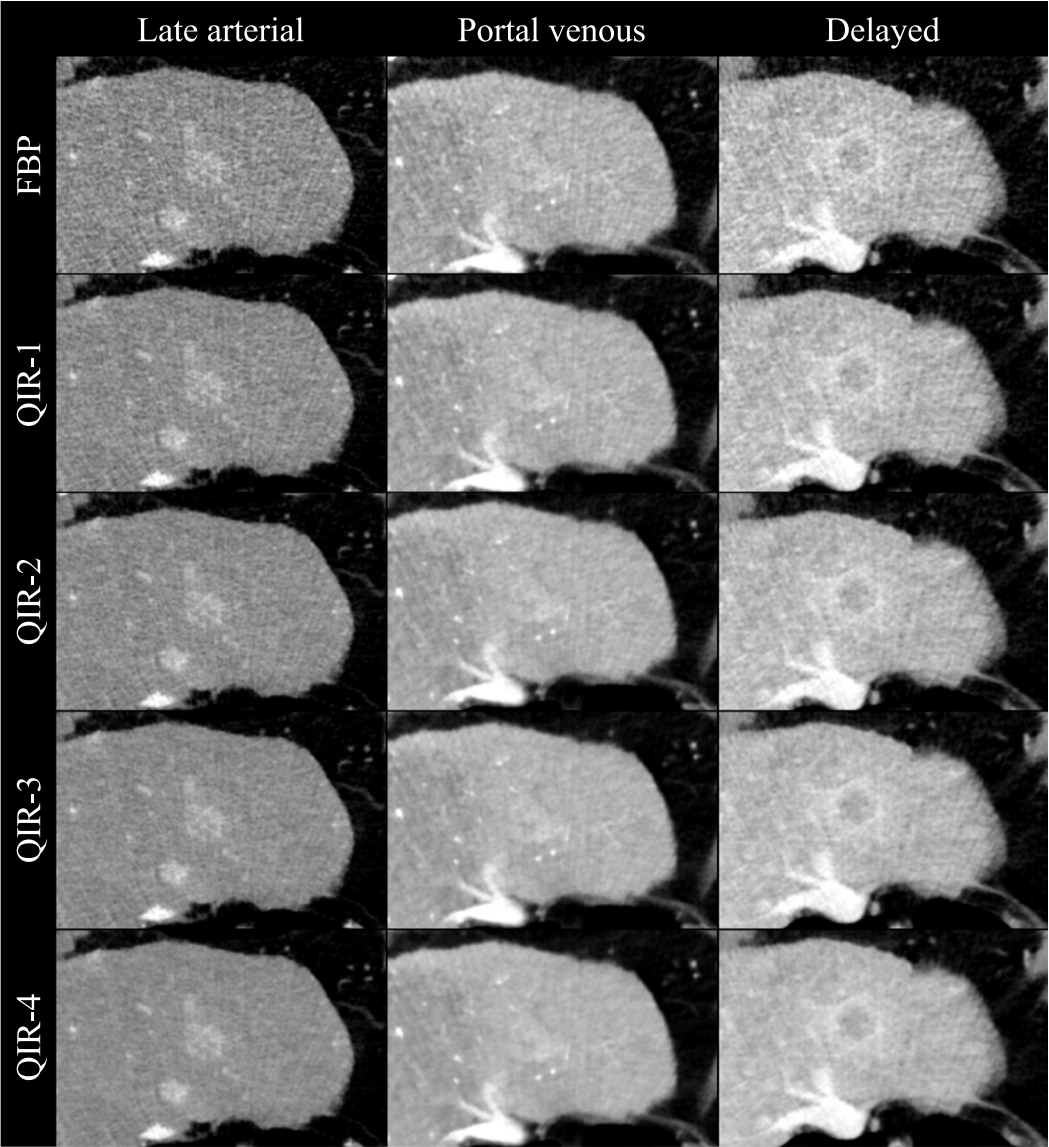
Set of example images. The images of the triple-phase HCC examination protocol reconstructed as filtered back projection and with the available quantum iterative reconstruction levels (QIR-1 to QIR-4). A typical HCC lesion is presented

Because high QIR-level images were subjectively assessed as not being relevantly disturbed by blurring effects, improvements in noise, artifacts, and lesion conspicuity consequently resulted in higher overall image quality scores. All differences in the quality criteria reached statistical significance (*p* < 0.05). A sample series of images illustrating the different QIR levels is shown in Fig. 4.

## Discussion

The results of this study show that the highest available QIR level provided the best image quality for diagnosing HCC lesions in triple-phase PCD-CT because of a noticeable noise reduction without quality losses from blurring.

The QIR algorithm was previously assessed for abdominal imaging in two studies by Racine et al. [31] and Sartoretti et al. [23]. Both groups reported improvement of liver lesion conspicuity by QIR using VMIs at 60 keV, Racine et al. in a pure phantom experiment and Sartoretti et al. in a heterogeneous study population for hypodense liver lesions in the portal venous phase. In contrast, the current work involved application of 50-keV VMIs of a triple-phase liver protocol for an HCC patient population. The higher iodine contrast at 50 keV is particularly important for HCC diagnosis, facilitating detection of hypervascular liver lesions in the late arterial phase and non-peripheral washout and enhancing capsule in the portal venous and delayed phases. As previously reported, keV levels ≤ 50 are impaired by increasing image noise [18, 21, 22]. QIR can compensate for this drawback, resulting in excellent image quality at the highest available level.

An alternative method for diagnosis of HCC is magnetic resonance imaging (MRI). Even though some studies have reported HCC imaging with MRI to be superior to energy-integrating detector (EID)-CT [32], a recent meta-analysis found no definitive superiority of EID-CT, extracellular contrast–enhanced MRI, or gadoxetate-enhanced MRI for HCC diagnosis in patients with cirrhosis [33]. Particularly, some factors limit application and image quality of MRI, including severe ascites, incapacity for longer breath holding, and claustrophobia. For these reasons, several studies have focused on optimization of triple-phase EID-CT, in which image quality improvements could be achieved with a combination of low tube voltage protocols with iterative reconstruction [34–37]. Even more quality improvements can be expected with PCD-CT, as a few previous studies have demonstrated for abdominal imaging. However, those studies were limited to only one contrast phase, i.e., either the late arterial phase [18] or the portal venous phase [21, 22]. In heterogeneous study populations, low-keV VMIs also have been discovered as yielding optimal images for diagnosis because of their distinct increase of iodine contrast.

The analyses in this study were focused on a cohort of patients with HCC. More generally, based on its characteristic signal behavior, HCC can be viewed as a pattern for hypervascular hepatic metastases, such as those of malignant melanoma and neuroendocrine tumors, and for hypovascular metastases, such as those of colorectal and lung cancer. In this sense, this work offers in vivo proof of the phantom study by Racine et al. [31].

This study has a few limitations. First, it was a single-center investigation with a limited sample size. Second, QIR was evaluated only for the softest quantitative reconstruction kernel (Qr36). Soft reconstruction kernels optimize CNR to the disadvantage of edge sharpness. Image sharpness of HCC lesions plays a minor role because of the intrinsic blurring of the lesions themselves. Nevertheless, images reconstructed using kernels with higher sharpness levels might be beneficial in specific cases, such as an optimized depiction of the intrahepatic arteries for planning transarterial chemoembolization. Previous studies of ultra-high-resolution coronary CT showed excellent image quality using a sharp kernel in combination with the highest QIR level (QIR-4) [38], and the feasibility of this approach for liver CT angiography should be investigated. Third, the median radiation doses are relatively high for abdominal scans. The primary focus in this HCC patient population was optimized image quality to achieve high diagnostic confidence. The potential for dose reduction in HCC PCD-CT imaging, as reported in a previous phantom study [29], needs to be confirmed in an in vivo study.

## Conclusions

In conclusion, the highest QIR level effectively suppresses noise in iodine contrast optimized low-keV VMIs and yields the best image quality for evaluating HCC in triple-phase PCD-CT. This approach might increase diagnostic accuracy and confidence in HCC imaging. Leveraging advanced spectral capabilities such as iodine quantification could lead to further improvements.

## Data Availability

The dataset used or analyzed during the current study are available from the corresponding author on reasonable request.

## Abbreviations

CNR: Contrast-to-noise ratio
CT: Computed tomography
CTDI: CT dose index
EID: Energy-integrating detector
FBP: Filtered back projection
HCC: Hepatocellular carcinoma
MRI: Magnetic resonance imaging
MTF: Modulation transfer function
NPS: Noise power spectrum
PCD: Photon-counting detector
QIR: Quantum iterative reconstruction
ROI: Region of interest
VMI: Virtual monoenergetic image
